# Primary analysis of a prospective cohort study of Japanese patients with plasma cell neoplasms in the novel drug era (2016–2021)

**DOI:** 10.1007/s12185-024-03754-8

**Published:** 2024-03-29

**Authors:** Hirohiko Shibayama, Mitsuhiro Itagaki, Hiroshi Handa, Akihiro Yokoyama, Akio Saito, Satoru Kosugi, Shuichi Ota, Makoto Yoshimitsu, Yasuhiro Tanaka, Shingo Kurahashi, Shin-ichi Fuchida, Masaki Iino, Takayuki Shimizu, Yukiyoshi Moriuchi, Kohtaro Toyama, Kinuko Mitani, Yutaka Tsukune, Akiko Kada, Hideto Tamura, Masahiro Abe, Hiromi Iwasaki, Junya Kuroda, Hiroyuki Takamatsu, Kazutaka Sunami, Masahiro Kizaki, Tadao Ishida, Toshiki Saito, Itaru Matsumura, Koichi Akashi, Shinsuke Iida

**Affiliations:** 1 Department of Hematology, NHO Osaka National Hospital, 2-1-14 Hoenzaka Chuo-ku, Osaka City, Osaka 540-0006 Japan; 2 Department of Hematology and Oncology, Osaka University Graduate School of Medicine, Suita, Japan; 3 Department of Hematology, Hiroshima Red Cross Hospital, Hiroshima, Japan; 4 Department of Hematology, Gunma University, Gunma, Japan; 5 Department of Hematology, NHO Tokyo Medical Center, Tokyo, Japan; 6 Department of Hematology, NHO Shibukawa Medical Center, Gunma, Japan; 7 Department of Hematology, Toyonaka Municipal Hospital, Osaka, Japan; 8 Department of Hematology, Sapporo Hokuyu Hospital, Hokkaido, Japan; 9 Department of Hematology and Rheumatology, Kagoshima University, Kagoshima, Japan; 10 Department of Hematology, Shinko Hospital, Hyogo, Japan; 11 Department of Hematology and Oncology, Toyohashi Municipal Hospital, Aichi, Japan; 12 Department of Hematology, JCHO Kyoto Kuramaguchi Medical Center, Kyoto, Japan; 13 Department of Hematology, Yamanashi Prefectural Central Hospital, Yamanashi, Japan; 14 Division of Hematology, Department of Medicine, Keio University School of Medicine, Tokyo, Japan; 15 Department of Hematology, Sasebo City General Hospital, Nagasaki, Japan; 16 Department of Hematology, Fujioka General Hospital, Gunma, Japan; 17 Department of Hematology and Oncology, Dokkyo Medical University, Tochigi, Japan; 18 Department of Hematology, Juntendo University School of Medicine, Tokyo, Japan; 19 Clinical Research Center, NHO Nagoya Medical Center, Aichi, Japan; 20 Division of Diabetes, Endocrinology and Hematology, Department of Internal Medicine, Dokkyo Medical University Saitama Medical Center, Saitama, Japan; 21 Department of Hematology, Kawashima Hospital, Tokushima, Japan; 22 Department of Hematology, NHO Kyushu Medical Center, Fukuoka, Japan; 23 Division of Hematology and Oncology, Department of Medicine, Kyoto Prefecture University of Medicine, Kyoto, Japan; 24 Department of Hematology, Kanazawa University, Kanazawa, Japan; 25 Department of Hematology, NHO Okayama Medical Center, Okayama, Japan; 26 Department of Hematology, Saitama Medical Center, Saitama Medical University, Saitama, Japan; 27 Department of Hematology, Japanese Red Cross Medical Center, Tokyo, Japan; 28 Department of Hematology and Rheumatology, Kindai University, Osaka, Japan; 29 Department of Medicine and Biosystemic Science, Kyushu University, Fukuoka, Japan; 30 Department of Hematology and Oncology, Nagoya City University Institute of Medical and Pharmaceutical Sciences, 1 Kawasumi, Mizuho-Cho, Mizuho-ku, Nagoya City, Aichi 467-8601 Japan

**Keywords:** Prospective cohort study, Plasma cell neoplasms (PCN), Three-year overall survival rate, Novel drug era

## Abstract

The emergence of novel drugs has significantly improved outcomes of patients with plasma cell neoplasms (PCN). The Japanese Society of Hematology conducted a prospective observational study in newly diagnosed PCN patients between 2016 and 2021. The analysis focused on 1385 patients diagnosed with symptomatic PCN between 2016 and 2018. The primary endpoint was the 3-year overall survival (OS) rate among patients requiring treatment (*n* = 1284), which was 70.0% (95%CI 67.4–72.6%). Approximately 94% of these patients received novel drugs as frontline therapy. The 3-year OS rate was 90.3% (95%CI 86.6–93.1%) in the 25% of patients who received upfront autologous stem cell transplantation (ASCT), versus just 61.4% (95%CI 58.0–64.6%) in those who did not receive upfront ASCT. The only unfavorable prognostic factor that affected OS in ASCT recipients was an age of 65 or higher. For patients who did not receive ASCT, independent unfavorable prognostic factors included frontline treatment with conventional chemotherapies, international staging system score of 2/3, extramedullary tumors, and Freiberg comorbidity index of 2/3. This study unequivocally demonstrates that use of novel drugs improved OS in Japanese myeloma patients, and underscores the continued importance of upfront ASCT as the standard of care in the era of novel drugs.

**Supplementary Information:**

The online version contains supplementary material available at 10.1007/s12185-024-03754-8.

## Introduction

According to data from the Japan National Cancer Registry, there were 7130 newly diagnosed multiple myeloma (NDMM) patients in 2015, comprising 3736 males and 3394 females. 4135 patients, consisting of 2030 males and 2105 females, passed away (1). The annual incidence rate of multiple myeloma gradually increased from 5.0 per 100,000 persons in 2010 to 6.0 in 2019 [1]. The estimated 5-year overall survival (OS) rate between 2009 and 2011 was 42.8% [1].

Multiple novel anti-myeloma drugs, including proteasome inhibitors (PIs), immunomodulatory drugs (IMiDs), and monoclonal antibodies (mAbs), have been developed and approved by the Pharmaceuticals and Medical Devices Agency (PMDA) for the treatment of multiple myeloma (MM) patients under the coverage of the national health insurance system in Japan. Initially, bortezomib was approved in 2006 for the treatment of relapsed and/or refractory multiple myeloma (RRMM). Subsequently, thalidomide and lenalidomide were approved for RRMM in 2008 and 2010, respectively. Based on the pivotal phase 3 VISTA [2] and FIRST [3] trials, bortezomib and lenalidomide were further approved for the treatment of newly diagnosed multiple myeloma (NDMM) in 2011 and 2015, respectively. In subsequent years, pomalidomide, carfilzomib and elotuzumab, ixazomib and daratumumab, and isatuximab were, respectively, approved for the treatment of RRMM in 2015, 2016, 2017, and 2020. Notably, daratumumab was also approved for the treatment of NDMM in 2019 based on the primary analyses of phase 3 trials, namely, ALCYONE [4, 5] and MAIA [6, 7].

Although the Japan National Cancer Registry operated by the National Cancer Center and the hematologic diseases registry operated by the Japanese Society of Hematology (JSH) exist, these registries do not gather sufficient data to conduct in-depth analyses of patient characteristics and outcomes. In 2015, Ozaki et al. provided a comprehensive overview of patient demographics and prognosis for Japanese multiple myeloma (MM) patients treated at hospitals where members of the Japanese Society of Myeloma (JSM) were employed [8]. The study retrospectively collected and analyzed real-world data from two cohorts: the 1990–2000 cohort consisting of 1208 patients and the 2001–2012 cohort consisting of 2234 patients, in collaboration with the JSM [8]. The analysis revealed a notable improvement in the 5-year overall survival (OS) rate, which increased from 31.2% in the 1990–2000 cohort to 50.3% in the 2001–2012 cohort. Furthermore, within the latter cohort, a subset of patients received treatment with novel drugs such as bortezomib, thalidomide, and lenalidomide, leading to a remarkable improvement in their prognosis. Since no national survey of Japanese MM patients has been conducted since the aforementioned report, we have undertaken a prospective collection and analysis of real-world data from the 2016–2018 cohort, in collaboration with the JSH, to elucidate the effects of novel anti-myeloma drugs.

## Methods

### Study design

The study described was a non-interventional, multicenter, prospective cohort study of the patients with plasma cell neoplasms (PCN) conducted in Japan, overseen by the Japanese Society of Hematology (UMIN000022099). The study adhered to the ethical principles outlined in the Declaration of Helsinki and the Ethical Guidelines for Medical and Health Research. The study protocol received approval from the institutional review boards (IRB) at all participating sites.

### Patients and treatments

A total of 67 sites across Japan participated in this study. Patients who were diagnosed with PCN between January 2016 and December 2018 were registered and followed up until the end of December 2021, patient withdrawal, or death. The PCN group encompassed both symptomatic conditions (such as symptomatic multiple myeloma, non-secretory multiple myeloma, multiple plasmacytoma, and plasma cell leukemia) and non-symptomatic conditions (such as monoclonal gammopathy of undetermined significance [MGUS], smoldering multiple myeloma, solitary plasmacytoma of bone, and extramedullary plasmacytoma), as per the diagnostic criteria of the International Myeloma Working Group (IMWG) [9, 10]. Patients with symptomatic PCN received treatment with drugs approved by the Pharmaceuticals and Medical Devices Agency (PMDA) based on the physician’s discretion.

### Endpoints

The primary endpoint of this study was the 3-year overall survival (OS) of newly diagnosed patients with symptomatic PCN who were treated with systemic chemotherapy. Secondary endpoints included progression-free survival (PFS), time to next treatment (TNT), treatment-free interval (TFI), and the best overall response rates (ORR) based on the International Myeloma Working Group (IMWG) uniform response criteria [11] as determined by the first-line treatment. The definitions of OS, PFS, TNT, and TFI can be found in Supplementary Method S1.

### Statistical analysis

In the United States, where novel anti-myeloma drugs were widely utilized, the real-world data showed a 3-year OS rate of 70% [12]. To achieve a 3-year survival rate with a 95% confidence interval (CI) range of less than 7%, a sample size of 800 patients with symptomatic PCN would be required. Accounting for non-symptomatic PCN and potential dropouts, the number of patients in this study was set at 1100. The analysis focused on patients diagnosed with symptomatic PCN who received treatment with anti-myeloma drugs, as well as patients initially diagnosed with non-symptomatic PCN at registration but progressed to symptomatic PCN requiring treatment during the study period. Baseline patient characteristics were summarized using descriptive statistics. PFS, OS, TNT, and TFI were estimated using the Kaplan–Meier method, and the 95% CI was calculated using the Greenwood formula. Univariable and multivariable Cox proportional hazards regression analyses were performed to assess the association between OS or PFS and clinically significant baseline factors, providing hazard ratios (HR) and their corresponding 95% CI. All statistical analyses were conducted using SAS version 9.4 (SAS Institute, Cary, NC, USA).

## Results

### Patient characteristics

**Fig. 1:**
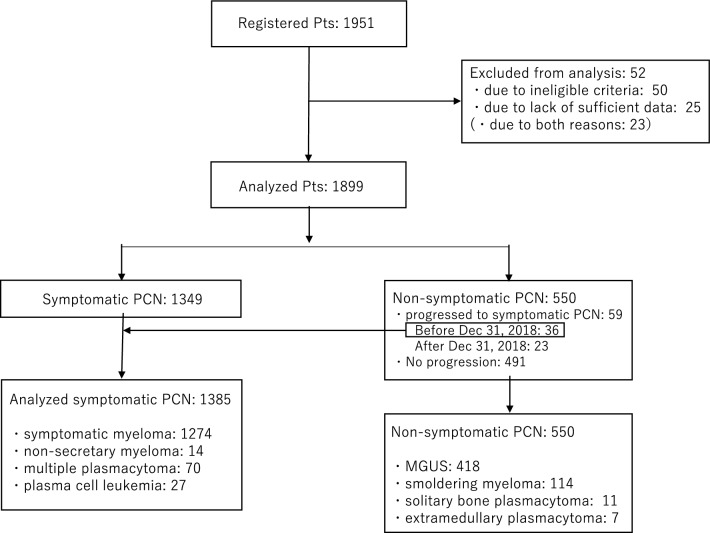
Registered patients were allocated as this branch shown here. A total of 1385 (1349 plus 36) symptomatic PCN patients were analyzed in this report

Between January 2016 and December 2018, a total of 1951 patients with PCN were registered from 67 hospitals across Japan. After excluding 52 patients due to ineligibility or insufficient data, 1899 patients were included in the analysis. Among them, 1349 patients were diagnosed with symptomatic PCN and required some form of treatment, while the remaining 550 patients were initially diagnosed with non-symptomatic PCN. During the study period, 59 patients with non-symptomatic PCN progressed to symptomatic PCN, with 36 of them experiencing this progression by December 2018. Therefore, a total of 1385 patients with symptomatic PCN (1349 plus 36) were analyzed in this report. Among these patients, 1274 were diagnosed with symptomatic multiple myeloma (MM), 14 with non-secretary MM, 70 with multiple plasmacytoma, and 27 with plasma cell leukemia (PCL) (Fig. 1).

In Table 1, the median age of the patients was 71 years (ranging from 33 to 96 years), and 1044 (75.4%) patients were 65 years or older. Female patients constituted 47.7% of the cohort. Eastern Cooperative Oncology Group Performance Status (ECOG PS) scores were distributed as 78.1% for 0–2 and 21.9% for 3–4. Regarding the ISS, 20.1%, 35.8%, and 40.1% of patients were classified into stages 1, 2, and 3, respectively. The M-protein types were IgG in 55.9% of cases and non-IgG in 40.4%. Various symptoms were observed in the patients, with hypercalcemia in 13.0%, renal insufficiency in 25.3%, anemia in 61.4%, and bone diseases in 61.5% of cases. Accompanying symptoms such as AL amyloidosis, extramedullary tumors, and central nervous system (CNS) invasion were observed in 6.1%, 8.4%, and 0.6% of the patients, respectively. The distribution of Freiburg comorbidity index (FCI) scores [13] was as follows: 61.7% had an FCI of 0, 29.2% had an FCI of 1, 7.9% had an FCI of 2, and 1.1% had an FCI of 3.

**Table 1:** Clinical characteristics of MM patients enrolled in this study

Baseline characteristics		Total (*n* = 1385)	
		*N*	%
Age, median (range)		71 (33–96)	
	0–64	341	24.6
	65–	1044	75.4
Sex	Male	725	52.3
	Female	660	47.7
ECOG PS	0	388	28.0
	1	476	34.4
	2	217	15.7
	3	195	14.1
	4	109	7.9
ISS	1	279	20.1
	2	496	35.8
	3	555	40.1
	Missing	55	4.0
M-protein type	IgG	774	55.9
	IgA	304	21.9
	IgD	34	2.5
	IgE	4	0.3
	IgM	7	0.5
	BJP	210	15.2
	Non-secretory	14	1.0
	Missing^a^	38	2.7
Myeloma defining events			
Hypercalcemia		180	13.0
Renal insufficiency		350	25.3
Anemia		850	61.4
Bone lesion		852	61.5
Myeloma related complications			
AL amyloidosis		85	6.1
Immunoglobulin deposition disease		26	1.9
Extramedullary tumor		116	8.4
CNS invasion		8	0.6
Past history			
Non-symptomatic PCN		100	7.2
Other malignancies than PCN		164	11.8
FCI (Freiburg comorbidity index)	0	855	61.7
	1	405	29.2
	2	110	7.9
	3	15	1.1
Diagnosis	Symptomatic multiple myeloma	1274	92.0
	Multiple plasmacytoma	70	5.1
	Plasma cell leukemia	27	1.9
	Non-secretory multiple myeloma	14	1.0

^a^Most of the patients whose M protein typing was not conducted by immunofixation or immuno-electrophoresis possessed measurable levels of free light chains of abnormal kappa/lambda ratios

In Table 2, we showed the clinical characteristics of the patients treated with and without autologous stem cell transplantation (ASCT). Nine-hundred and twenty-two patients received the initial induction therapy but did not undergo ASCT (non-ASCT-Group), while the remining 342 patients underwent ASCT within 1 year from the start of the initial induction therapy (ASCT-Group). The patients who received no initial induction therapies (*n* = 101), that who received an allogeneic transplant (*n* = 1), or those who underwent ASCT after 1 year from the start date of the initial therapy (*n* = 19) were not included in either group. The median ages (range) were 75 (37–92) in non-ASCT-G and 60 (33–73) in ASCT-G, respectively. 91% and 31.9% of the patients of non-ASCT-G and ASCT-G were over 65 years. ECOG PS 0–2/3–4 in non-ASCT-G and in ASCT-G were 77.7/22.3% and 85.4/14.6%, respectively. ISS 1/2/3 in non-ASCT-G and in ASCT-G were 14.9/35.8/44.9% and 35.4/36/27.5%, respectively. The symptoms of hypercalcemia, renal insufficiency, anemia, and bone diseases were seen in 13.3, 28.4, 65.3, and 58% of non-ASCT-G and in 11.4, 16.4, 51.8, and 75.4% of ASCT-G. FCI 0/1/2/3 in non-ASCT-G and in ASCT-G were 57.9/32.2/8.8/1.1% and 76.6/20.2/2.6/0.6%, respectively.

**Table 2:** Clinical characteristics of MM patients with and without autologous stem cell transplantation

Baseline characteristics		Non-ASCT (*n* = 922)^a^		ASCT (*n* = 342)^b^	
		*N*	%	*N*	%
Age, median (range)		75 (37–92)		60 (33–73)	
	0–64	83	9.0	233	68.1
	65–	839	91.0	109	31.9
Sex	Male	462	50.1	192	56.1
	Female	460	49.9	150	43.9
ECOG PS	0	225	24.4	138	40.4
	1	337	36.6	111	32.5
	2	154	16.7	43	12.6
	3	139	15.1	32	9.4
	4	67	7.3	18	5.3
ISS	1	137	14.9	121	35.4
	2	330	35.8	123	36.0
	3	414	44.9	94	27.5
	Missing	41	4.4	4	1.2
M-protein type	IgG	530	57.5	175	51.2
	IgA	205	22.2	74	21.6
	IgD	23	2.5	9	2.6
	IgE	3	0.3	1	0.3
	IgM	6	0.7	0	0.0
	BJP	127	13.8	67	19.6
	Non-secretory/missing	28	3.0	16	4.7
Myeloma defining events					
Hypercalcemia		123	13.3	39	11.4
Renal insufficiency		262	28.4	56	16.4
Anemia		602	65.3	177	51.8
Bone lesion		535	58.0	258	75.4
Myeloma related complications					
AL amyloidosis		61	6.6	12	3.5
Immunoglobulin deposition disease		19	2.1	4	1.2
Extramedullary tumor		75	8.1	28	8.2
CNS invasion		7	0.8	1	0.3
Past history					
Non-symptomatic PCN		71	7.7	23	6.7
Other malignancies than PCN		124	13.4	20	5.8
FCI (Freiburg comorbidity index)	0	534	57.9	262	76.6
	1	297	32.2	69	20.2
	2	81	8.8	9	2.6
	3	10	1.1	2	0.6
Diagnosis	Symptomatic multiple myeloma	847	91.9	313	91.5
	Multiple plasmacytoma	46	5.0	17	5.0
	Plasma cell leukemia	19	2.1	8	2.3
	Non-secretory multiple myeloma	10	1.1	4	1.2

Patients who received no initial treatment (*n* = 101), that who received an allogeneic stem cell transplant (*n* = 1), or those who received an autologous transplant after 1 year of initial treatment (*n* = 19) were not included in either group

^a^Patients who received initial induction therapy without any transplant

^b^Patients who underwent autologous stem cell transplant within 1 year of initial induction therapy

### Overall survival and its prognostic factors

**Fig. 2:**
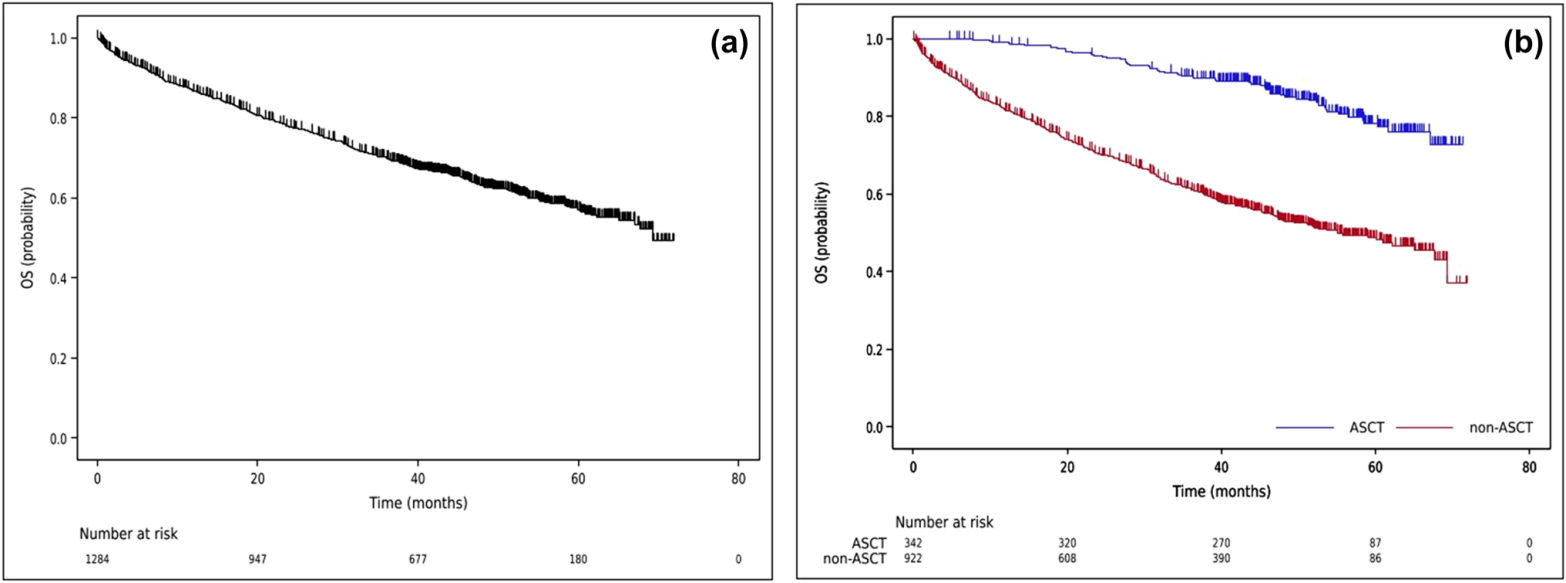
Survival curves of the symptomatic PCN patients who needed any kinds of treatments (*n* = 1284) are shown. **a** Three-year overall survival (OS) rate was 70.0% (95%CI 67.4–72.6%). The median OS period was 69.3 months (95%CI 67.1-NE). **b** Three-year OS rates of the patients who received upfront ASCT (*n* = 342, blue line) and who did not receive ASCT as initial therapies (*n* = 922, red line) were 90.3% (95%CI 86.6–93.1%) and 61.4% (95%CI 58.0–64.6%), respectively. The median OS periods of the former and the latter patient groups were NE and 55.1 months (95%CI 47.4–67.7), respectively

The 3-year overall survival (OS) rate for symptomatic patients with PCN who required any form of treatment (*n* = 1284) was found to be 70.0% with a 95% CI of 67.4% to 72.6% (Fig. 2a). Among these patients, 342 individuals (24.7%) received upfront ASCT, while 922 patients (66.6%) did not undergo ASCT as their initial therapy but received conventional treatments. In subgroup analysis, the 3-year OS rates for the former and latter patient groups were 90.3% (95%CI 86.6–93.1%) and 61.4% (95%CI 58.0–64.6%), respectively (Fig. 2b). Tables 3 and 4 illustrate the results of further analysis. In the patient group who received ASCT, only age 65 or older had a significant impact on worse OS rate, with a HR of 2.24 (95%CI 1.33–3.77) in multivariable analysis (Suppl Fig. 1a). On the other hand, in the patient group who did not undergo ASCT, induction treatments containing immunomodulatory drugs (IMiD), proteasome inhibitors (PI), and the combination of IMiD and PI were associated with better OS rates compared to conventional chemotherapy, with HRs of 0.64 (95%CI 0.43–0.95), 0.69 (95%CI 0.48–1.00), and 0.59 (95%CI 0.37–0.93), respectively, as determined by multivariable analysis. Furthermore, ISS 2 and ISS 3 were associated with worse OS rates compared to ISS 1, with HRs of 2.05 (95%CI 1.38–3.02) and 2.82 (95%CI 1.91–4.19), respectively (Suppl Fig. 1b). Similarly, patients with extramedullary tumors exhibited a worse OS rate, with a HR of 2.00 (95%CI 1.46–2.76) (Suppl Fig. 1c). In addition, patients with an FCI of 2/3 had a worse OS rate compared to those with an FCI of 0, with an HR of 1.98 (95%CI 1.43–2.72) (Suppl Fig. 1d).

**Table 3:** Univariable and multivariable analyses for OS in MM patients receiving ASCT

Variables	Univariable			Multivariable		
	HR	95%CI	*p* value	HR	95%CI	*p* value
Age 65 or older	2.03	1.22–3.37	0.006	2.24	1.33–3.77	0.002
Sex, male	1.72	1.00–2.94	0.049	1.67	0.96–2.89	0.069
ISS stage 2^a^	1.94	1.00–3.77	0.052	1.89	0.97–3.69	0.063
ISS stage 3^a^	2.05	1.01–4.16	0.046	1.98	0.90–4.35	0.088
Creatinine ≤ 2 mg/dL	0.96	0.41–2.24	0.933			
Presence of extramedullary tumor	1.01	0.41–2.53	0.979	0.99	0.39–2.52	0.979
CA, del(17p)	2.72	1.43–5.15	0.002			
CA, t(4;14)	1.40	0.71–2.75	0.336			
CA, t(14;16)	4.72	2.23–9.99	< 0.001			
CA, t(11;14)	0.58	0.17–1.97	0.384			
CA, del(13) or del(13q)	1.90	0.80–4.52	0.146			
CA, hypodiploidy	1.33	0.51–3.47	0.556			
CA, any	2.97	1.68–5.25	< 0.001			
FCI, 1^b^	1.10	0.59–2.05	0.762	0.91	0.46–1.79	0.778
FCI, 2–3^b^	2.46	0.88–6.87	0.085	1.29	0.36–4.70	0.695

Each cytogenetic abnormality was not applied for multivariable analysis, as it was examined only in a fraction of the patients

ISS: International Staging System; CA: chromosomal abnormalities; FCI: Freiburg Comorbidity Index; HR: hazard ratio; CI: confidence interval

^a^ISS stage 1 is reference category

^b^FCI 0 is reference category

**Table 4:** Univariable and multivariable analyses for OS in MM patients not receiving ASCT

Variables	Univariable			Multivariable		
	HR	95%CI	*p* value	HR	95%CI	*p* value
IMiD-based induction therapy	0.51	0.35–0.74	0.001	0.64	0.43–0.95	0.029
PI-based induction therapy	0.75	0.53–1.07	0.113	0.69	0.48–1.00	0.048
PI plus IMiD-based Induction therapy	0.52	0.34–0.80	0.003	0.59	0.37–0.93	0.023
Age 65 or older	0.98	0.70–1.38	0.914	0.99	0.69–1.41	0.944
Sex, male	1.28	1.05–1.56	0.015	1.21	0.99–1.48	0.062
ISS stage 2^a^	2.05	1.39–3.02	< 0.001	2.05	1.38–3.02	< 0.001
ISS stage 3^a^	3.39	2.34–4.92	< 0.001	2.82	1.91–4.19	< 0.001
Creatinine ≤ 2 mg/dL	1.57	1.25–1.98	< 0.001			
Presence of extramedullary tumor	1.87	1.38–2.54	< 0.001	2.00	1.46–2.76	< 0.001
CA, del(17p)	1.66	1.19–2.32	0.003			
CA, t(4;14)	1.29	0.96–1.75	0.095			
CA, t(14;16)	1.62	0.99–2.65	0.057			
CA, t(11;14)	1.07	0.74–1.54	0.723			
CA, del(13) or del(13q)	1.35	0.92–1.98	0.121			
CA, hypodiploidy	1.44	0.93–2.23	0.102			
CA, any	1.44	1.13–1.85	0.004			
FCI, 1^b^	1.55	1.25–1.93	< 0.001	1.18	0.94–1.50	0.160
FCI, 2-3^b^	3.15	2.37–4.18	< 0.001	1.98	1.43–2.72	< 0.001

Each cytogenetic abnormality was not applied for multivariable analysis, as it was examined only in a fraction of the patients

IMiD: immunomodulatory drug; PI: proteasome inhibitor: ISS: International Staging System; CA: chromosomal abnormalities; FCI: Freiburg Comorbidity Index; HR: hazard ratio; CI: confidence interval

^a^ISS stage 1 is reference category

^b^FCI 0 is reference category

### Response rates

**Fig. 3:**
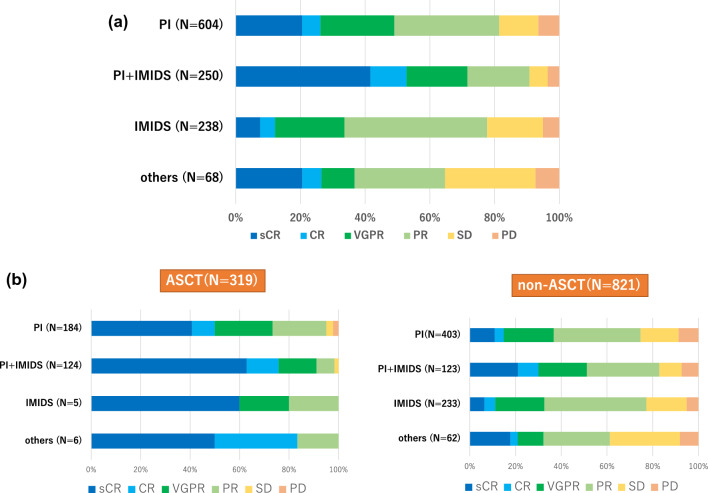
Best responses according to the first line therapies are shown. **a** All patients, **b** patients who received upfront ASCT (ASCT) and those who did not receive ASCT (non-ASCT)

As first-line therapies, 604 patients received PI, 250 patients received a combination of PI and IMiD, 238 patients received IMiD alone, and 68 patients received conventional chemotherapies. Regarding overall response rates, including partial response (PR) or better and very good PR or better, the combination of PI and IMiD exhibited the highest rate (Fig. 3a). Notably, in both patient groups, those who received ASCT and those who did not receive ASCT, the combination regimens involving both PI and IMiD as the first-line therapy demonstrated better response rates compared to PI-based or IMiD-based regimens (Fig. 3b). Furthermore, ASCT-G showed higher rates of deeper responses represented by very good PR (VGPR) or better than non-ASCT-G regardless of the kinds of the induction regimens (Fig. 3b).

### Progression free survival (PFS) and its prognostic factors

**Fig. 4:**
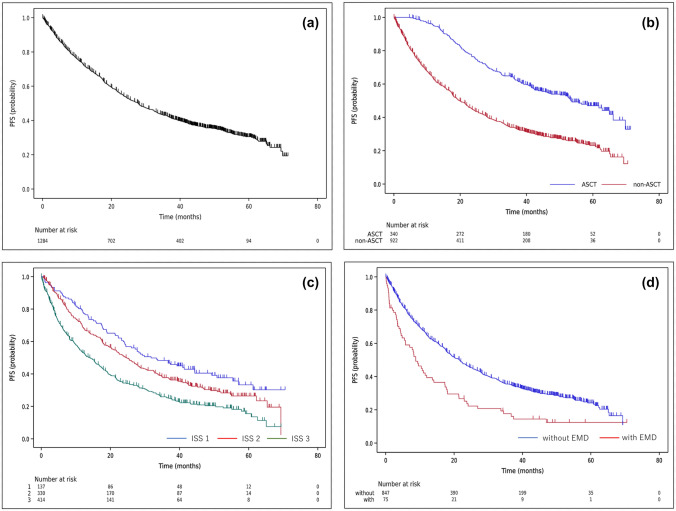
Progression free survival (PFS) curves of the symptomatic PCN patients are shown. **a** Three-year PFS rate was 43.0% (95%CI 40.2–45.8%). The median PFS period was 27.8 months (95%CI 24.8–30.4). **b** Three-year PFS rates of ASCT patients (blue line) and non-ASCT patients (red line) were 64.3% (95%CI 58.8–69.2%) and 34.0% (95%CI 30.8–37.3%), respectively. The median PFS periods of the former and the latter patient groups were 53.4 months (95%CI 44.6–65.0) and 19.9 months (95%CI 17.7–22.3), respectively. Two patients receiving ASCT who showed PD before ASCT were excluded from this PFS analysis. **c** Three-year PFS rate of non-ASCT patients with ISS 3 (green line) was significantly worse than that with ISS 1 (blue line). **d** Three-year PFS rate of non-ASCT patients with extramedullary tumors (EMD) (red line) was significantly worse than that without extramedullary tumors (blue line)

The 3-year progression-free survival (PFS) rate, which is one of the secondary endpoints of this study, was found to be 43.0% with a 95% CI of 40.2% to 45.8% (Fig. 4a). Among patients who received ASCT and those who did not, the 3-year PFS rates were 64.3% (95%CI 58.8–69.2%) and 34.0% (95%CI 30.8–37.3%), respectively (Fig. 4b). At a median follow-up of 4.5 years, the median PFS for all symptomatic PCN patients was 27.8 months (95%CI 24.8–30.4). In the ASCT group, the median PFS was 53.4 months (95%CI 44.6–65.0), whereas in the non-ASCT group, it was 19.9 months (95%CI 17.7–22.3). Tables 5 and 6 present the results of multivariable analysis. In the patient group who received ASCT, no factors were significantly associated with PFS. However, in the patient group who did not undergo ASCT, induction treatment with IMiD was associated with better PFS compared to conventional chemotherapy, with an HR of 0.70 (95%CI 0.50–0.97). As unfavorable prognostic factors, ISS 3 was found to be associated with poorer PFS compared to ISS 1, with an HR of 1.83 (95%CI 1.40–2.38) (Fig. 4c). In addition, the presence of extramedullary tumors was significantly associated with a worse PFS rate, with an HR of 1.87 (95%CI 1.42–2.46) (Fig. 4d).

**Table 5:** Univariable and multivariable analyses for PFS in MM patients receiving ASCT

Variables	Univariable			Multivariable		
	HR	95%CI	p value	HR	95%CI	p value
Age 65 or older	1.25	0.91–1.73	0.169	1.30	0.94–1.80	0.116
Sex, male	1.39	1.02–1.91	0.039	1.34	0.97–1.85	0.073
ISS stage 2^a^	1.31	0.91–1.89	0.153	1.31	0.90–1.90	0.154
ISS stage 3^a^	1.45	0.98–2.16	0.063	1.43	0.93–2.22	0.107
ECOG PS 2-4^b^	1.26	0.91–1.75	0.171			
Creatinine ≤ 2 mg/dL	0.92	0.55–1.54	0.752			
Presence of extramedullary tumor	1.28	0.75–2.18	0.364	1.26	0.73–2.17	0.398
CA, del(13) or del(13q)	1.42	0.78–2.60	0.248			
CA, del(17p)	2.03	1.28–3.23	0.003			
CA, t(4;14)	1.62	1.07–2.46	0.021			
CA, t(14;16)	1.80	0.93–3.45	0.079			
CA, t(11;14)	0.90	0.47–1.71	0.748			
CA, hypodiploidy	1.19	0.61–2.32	0.617			
CA, any	1.83	1.28–2.60	0.001			
FCI, 1^c^	0.99	0.67–1.45	0.960	0.90	0.60–1.35	0.605
FCI, 2-3^c^	1.99	0.97–4.06	0.060	1.42	0.62–3.26	0.406

Each cytogenetic abnormality was not applied for multivariable analysis, as it was examined only in a fraction of the patients

IMID: immunomodulatory drug; PI: proteasome inhibitor: ISS: International Staging System; CA: chromosomal abnormalities; FCI: Freiburg Comorbidity Index; HR: hazard ratio; CI: confidence interval

^a^ISS stage 1 is reference category

^b^ECOG PS 0–1 is reference category

^c^FCI 0 is reference category

**Table 6:** Univariable and multivariable analyses for PFS in MM patients not receiving ASCT

Variables	Univariable			Multivariable		
	HR	95%CI	*p* value	HR	95%CI	*p* value
IMiD-based induction therapy	0.62	0.45–0.86	0.004	0.70	0.50–0.97	0.035
PI-based induction therapy	0.94	0.69–1.27	0.675	0.83	0.61–1.14	0.247
PI plus IMiD-based induction therapy	0.82	0.57–1.16	0.260	0.90	0.62–1.30	0.561
Age 65 or older	1.00	0.76–1.32	0.992	0.96	0.72–1.29	0.797
Sex, male	1.13	0.96–1.32	0.130	1.10	0.93–1.29	0.267
ISS stage 2^a^	1.30	1.00–1.67	0.046	1.29	1.00–1.67	0.050
ISS stage 3^a^	1.95	1.53–2.50	< 0.001	1.83	1.40–2.38	< 0.001
ECOG PS 2-4^b^	1.38	1.18–1.62	< 0.001			
Creatinine, ≤ 2 mg/dL	1.48	1.22–1.80	< 0.001			
Presence of extramedullary tumor	1.82	1.40–2.36	< 0.001	1.87	1.42–2.46	< 0.001
CA, del(13) or del(13q)	1.24	0.89–1.73	0.211			
CA, del(17p)	1.46	1.09–1.95	0.010			
CA, t(4;14)	1.39	1.09–1.78	0.009			
CA, t(14;16)	1.32	0.86–2.04	0.206			
CA, t(11;14)	1.28	0.95–1.72	0.104			
CA, hypodiploidy	1.34	0.91–1.97	0.132			
CA, any	1.48	1.21–1.81	< 0.001			
FCI, 1^c^	1.32	1.11–1.56	0.002	1.10	0.91–1.33	0.308
FCI, 2-3^c^	1.92	1.49–2.49	< 0.001	1.29	0.97–1.71	0.084

Each cytogenetic abnormality was not applied for multivariable analysis, as it was examined only in a fraction of the patients

IMiD: immunomodulatory drug; PI: proteasome inhibitor; ISS: International staging system; CA: chromosomal abnormalities; FCI: Freiburg Comorbidity Index; HR: hazard ratio; CI: confidence interval

^a^ISS stage 1 is reference category

^b^ECOG PS 0–1 is reference category

^c^FCI 0 is reference category

### OS and PFS analyses according to the cytogenetic risk category and revised-ISS (R-ISS)[14]

Among the 284 patients who received ASCT and had available baseline fluorescence in situ hybridization (FISH) data, the 3-year overall survival (OS) rate for 84 patients with high-risk cytogenetics carrying either t(4;14), t(14;16), or del(17p) was 84.3% with a 95% CI of 74.6% to 90.6%. In contrast, the 3-year OS rate for 200 patients with standard-risk cytogenetics was 93.2% (95%CI 88.6–96.0) (Suppl Fig. 2a). For the 654 patients who did not receive ASCT and had available baseline FISH data, the 3-year OS rate and median OS for 164 patients with high-risk cytogenetics were 49.7% (95%CI 41.6–57.2) and 35.4 months (95%CI 29.7–46.9), respectively. In comparison, the 3-year OS rate and median OS for 490 patients with standard-risk cytogenetics were 65.2% (95%CI 60.6–69.4) and 60.9 months (95%CI 49.4-NA) (Suppl Fig. 2b). Among the 256 patients who received ASCT and were categorized into revised International Staging System (ISS) based on baseline data, the 3-year OS rates were 92.9% (95%CI 82.1–97.3), 89.4% (95%CI 83.5–93.3), and 87.9% (95%CI 70.9–95.3) for stages 1, 2, and 3, respectively (Suppl Fig. 2c). For the 565 patients who did not receive ASCT and were categorized into revised ISS stages based on baseline data, the 3-year OS rates were 88.1% (95%CI 76.5–94.1), 64.7% (95%CI 59.2–69.6), and 41.4% (95%CI 33.2–49.5) for stages 1, 2, and 3, respectively (Suppl Fig. 2d).

Among the 282 patients who received ASCT and had available baseline FISH data, the 3-year PFS rate and median PFS for 83 patients with high-risk cytogenetics were 52.4% (95%CI 41.1–62.6) and 36.6 months (95%CI 26.8–55.7), respectively. In contrast, the 3-year PFS rate and median PFS for 199 patients with standard-risk cytogenetics were 72.8% (95%CI 65.9–78.6) and 65.0 months (95%CI 53.3-NA), respectively (Suppl Fig. 2e). For the 654 patients who did not receive ASCT and had available baseline FISH data, the 3-year PFS rate and median PFS for 164 patients with high-risk cytogenetics were 20.9% (95%CI 15.0–27.6) and 15.2 months (95%CI 11.8–17.5), respectively. In comparison, the 3-year PFS rate and median PFS for 490 patients with standard-risk cytogenetics were 38.6% (95%CI 34.1–43.1) and 23.5 months (95%CI 19.5–27.9), respectively (Suppl Fig. 2f).

### Time to next treatment (TNT) and treatment free interval (TFI)

**Fig. 5:**
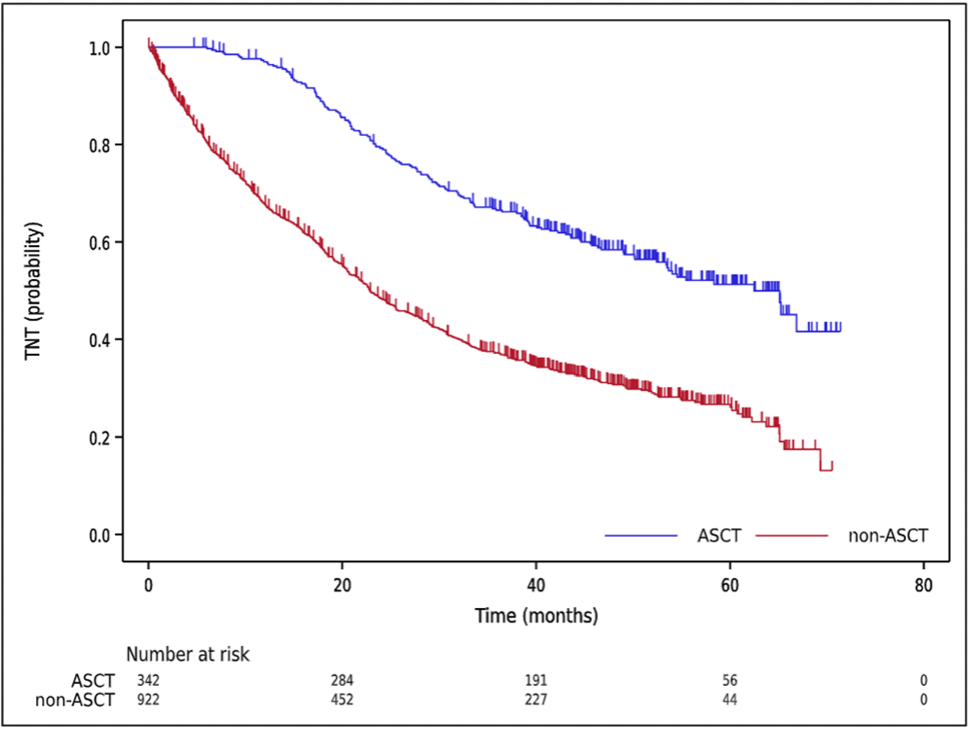
Time to next treatment (TNT) curves of the symptomatic PCN patients are shown. The median TNT of ASCT (blue line) and non-ASCT (red line) were 62.5 months (95% CI 53.4–NA) and 22.8 months (95% CI 20.7–25.5), respectively

The median TNT for all symptomatic patients with PCN was 30.9 months with a 95% CI of 28.7 to 33.7 months. Among patients who received ASCT, the median TNT was 62.5 months (95%CI 53.4-NA), whereas for those who did not receive ASCT, it was 22.8 months (95%CI 20.7–25.5) (Fig. 5).

The median TFI for all symptomatic PCN patients was 1.2 months with a 95% CI of 1.0 to 1.4 months. Among patients who received ASCT, the median TFI was 2.8 months (95%CI 2.1–8.8), while for those who did not receive ASCT, it was 1.0 month (95%CI 0.8–1.1) (data not shown).

## Discussion

In this prospective observational study conducted in Japan from 2016 to 2018, the overall survival (OS) rate of patients with PCN was found to be 70.0% at 3 years. This OS rate is consistent with data reported by Kumar SK, et al. from Mayo Clinic, Rochester, MN, USA [12]. In the Mayo Clinic study, they analyzed 1038 patients with newly diagnosed multiple myeloma (MM) between 2001 and 2010, grouping them into two 5-year periods: the 2001–2005 cohort and the 2006–2010 cohort. The OS rate at 3 years was 65% in the 2001–2005 cohort and 72% in the 2006–2010 cohort. Furthermore, the Mayo Clinic study also showed that the 3-year OS rate was 77% in patients receiving one of the novel drugs, such as thalidomide, lenalidomide, or bortezomib, as their initial therapy, compared to 67% in patients who did not receive these drugs. Similarly, in our cohort, until the approval of daratumumab for newly diagnosed MM in 2019, only bortezomib and lenalidomide among the novel drugs were approved for the patients with newly diagnosed MM. As a result, approximately 94% of the patients in our cohort received bortezomib and/or lenalidomide as their initial therapy, which contributed to achieving similar outcomes to that of the Mayo Clinic cohort in the 2006–2010 period. This study clearly demonstrates that the use of novel drugs has significantly improved the outcomes of Japanese patients with PCN.

The previous retrospective study of the real-world outcomes of Japanese patients with symptomatic MM reported by S. Ozaki et al. showed that the 3-year OS rates improved from 55% in the 1990–2000 cohort to 70% in the 2001–2012 cohort [8]. They also demonstrated that the 3-year OS rates were 70% in the patients receiving the novel drugs and 58% in the patients receiving the conventional chemotherapies alone in 2001–2012 cohort. In our current prospective study (2016–2018 cohort), we found a 3-year OS rate of 70.0% for patients with symptomatic PCN who received any kinds of treatment. Although there were differences in the median age (67 years and 71 years, respectively) and rates of patients receiving ASCT (32.3% and 27.1%, respectively) between the previous study and our current study, the 3-year OS rates at 3 years were similar. However, it is important to note that our current study enrolled patients prospectively from various kinds of hospitals, including those with general hematologists belonging to the Japanese Society of Hematology (JSH), representing a broader real-world population of symptomatic PCN patients. Based on this perspective, our current study’s findings suggest that the real-world outcomes of MM treatment have continued to improve further in 2016–2018 compared to the previous period (2001–2012). This improvement may be attributed to the wider availability and use of novel drugs in the treatment of symptomatic PCN patients during this study period.

Our study showed that the OS, PFS rates and TNT of patients who received ASCT showed better outcome. It possibly results from that many patients in ASCT-G achieved deeper responses. The DETERMINATION study’s results, which compared continuous therapy with a novel drug triplet regimen containing bortezomib, lenalidomide, and dexamethasone with upfront ASCT, demonstrated that continuous therapy did not show superior progression-free survival (PFS) despite showing similar overall survival (OS) [15]. This finding suggests that upfront ASCT remains a standard of care for transplant-eligible (TE) patients. On the other hand, daratumumab-containing therapies (D-MPB [daratumumab, melphalan, prednisolone plus bortezomib] and D-Ld [daratumumab, lenalidomide plus dexamethasone]) have shown more favorable PFS in transplant-ineligible (TIE) patients in the ALCYONE and MAIA studies [4, 6]. As these therapies become more widely used, the role of upfront ASCT may become less significant in the near future. The next prospective observational cohort study (JSH-MM-20) that started in Japan in January 2022 will provide further insights into the use of daratumumab-containing therapies in newly diagnosed MM patients and will help clarify the role of upfront ASCT in the context of evolving treatment options. Overall, ongoing research and clinical trials will continue to shape the treatment landscape for MM patients, and the role of upfront ASCT will be better understood in the context of novel drug therapies and patient selection criteria.

This study has revealed valuable insights into the factors influencing OS and PFS in patients with PCN, particularly those who did or did not receive upfront ASCT. Currently, upfront ASCT is one of the alternative choices for the patients aged 65–70 years as reported by a retrospective study conducted by Japanese Society of Myeloma and European Myeloma Network [16]. However, this study showed in patients who received upfront ASCT, the primary factor affecting OS was age. Specifically, the study found that patients aged 65 or older had an unfavorable OS, highlighting the need for careful patient selection when considering upfront ASCT, especially in elderly patients. This finding emphasizes the importance of considering age-related factors and potential treatment-related risks when deciding on treatment strategies for older MM patients. Conversely, in patients who did not receive ASCT, several risk factors were associated with unfavorable outcomes, including the treatment regimen of conventional chemotherapies, ISS stages 2/3, the presence of extramedullary tumors, and FCI stages 2/3. These factors did not include age in the group of patients who did not receive ASCT, indicating that other clinical factors play a more significant role in predicting OS in this subgroup. The study’s findings have important implications for clinical decision-making, especially when considering upfront ASCT as an alternative treatment choice for elderly patients, particularly those aged at 65 or older. The results underscore the need for a personalized and comprehensive assessment of individual patient characteristics, including age, comorbidities, disease stage, and treatment response, when determining the appropriateness of upfront ASCT. This approach ensures that the potential benefits of ASCT are balanced against the individual patient’s overall health status and potential risk factors.

It was confirmed that the use of novel drugs, particularly bortezomib and lenalidomide, as initial therapies has led to better ORR and improved OS compared to conventional chemotherapies in non-ASCT group. This improvement is noteworthy, even though the number of patients treated with conventional chemotherapies was limited. Among the novel drug options, the combination of bortezomib and lenalidomide emerged as particularly effective, as evidenced by the highest ORR and a deeper response with VGPR or higher. In non-ASCT group, it was observed that factors such as ISS stage 3 and the presence of extramedullary tumors were associated with unfavorable OS and PFS even in the era of novel drugs. This suggests that the current treatment approaches with bortezomib and/or lenalidomide may not be sufficient for these specific high-risk TIE patients. This highlights the need for alternative treatment strategies, such as incorporating daratumumab-containing therapies, such as D-MPB or D-Ld, which have shown more favorable results in the context of high-risk MM patients. In addition, the study’s findings emphasize that it’s not just chronological age, but comorbidities, that significantly influence OS in TIE patients. This suggests that older patients who are otherwise fit and without significant comorbidities can still benefit from bortezomib and/or lenalidomide-based regimens. Individualized treatment decisions based on a patient’s overall health status, beyond just age, are crucial for optimizing outcomes. While the study did not conduct a multivariable analysis for baseline prognostic factors related to FISH analyses due to the small number of patients, the mention of high-risk cytogenetics (such as t(4;14), t(14;16), or del(17p)) aligns with previous reports that these cytogenetic abnormalities can negatively impact both PFS and OS [17, 18]. This underscores the importance of developing novel immune-based therapies, such as chimeric antigen receptor-T (CAR-T) cell therapy and bispecific antibodies, to address these high-risk cases and further improve treatment outcomes [19, 20]. Overall, this study identifies several areas where additional research and novel therapeutic approaches are needed to address the specific challenges faced by high-risk TIE patients with MM. This underscores the ongoing unmet need to improve treatment strategies for this patient population.

It’s clear that while this study provides valuable insights into the outcomes of patients with PCN, there are certain limitations that should be considered. One significant limitation is the lack of comprehensive safety data beyond the occurrence of second primary malignancies. Detailed safety data, adverse events, and the relative dose intensities of the drugs used could provide a more complete picture of the treatment landscape and potential challenges faced by patients. The observational nature of this study, with treatment decisions being based on physicians’ choice and the availability of national health insurance, introduces variability in the treatments and examinations received by patients. This variability can impact the comprehensiveness of data collected, such as the limited availability of baseline chromosomal analysis like FISH, which in turn affects the ability to conduct multivariable analyses. Despite these limitations, the study’s reliance on real-world data collected from a national survey is still highly valuable. This type of data is particularly important in rapidly evolving fields like multiple myeloma, where treatment strategies are continually changing due to the emergence of new therapies. Real-world data can provide insights into the effectiveness of these treatments in actual clinical practice, and it also enables international comparisons. The commitment to continuing this type of survey in collaboration with the Japanese Society of Hematology demonstrates a dedication to advancing the understanding of plasma cell neoplasms and improving patient outcomes. The approach of using real-world data to identify unresolved clinical questions and guide future research is an essential step toward refining treatment strategies and ultimately enhancing the prognosis of patients with these conditions.

### Supplementary Information

Below is the link to the electronic supplementary material.

Supplementary file 1 (PPTX 486 KB)

Supplementary file 2 (DOCX 16 KB)

## References

[1] The latest statistics of the data from the Japan national cancer registry. http://ganjoho.jp/public/statistics/

[2] Mateos MV, Richardson PG, Schlag R, Khuageva NK, Dimopoulos MA, Shpilberg O, Kropff M, Spicka I, Petrucci MT, Palumbo A, Samoilova OS, Dmoszynska A, Abdulkadyrov KM, Schots R, Jiang B, Esseltine DL, Liu K, Cakana A, van de Velde H, San Miguel JF (2010). Bortezomib plus melphalan and prednisone compared with melphalan and prednisone in previously untreated multiple myeloma: updated follow-up and impact of subsequent therapy in the phase III VISTA trial. J Clin Oncol.

[3] Benboubker L, Dimopoulos MA, Dispenzieri A, Catalano J, Belch AR, Cavo M, Pinto A, Weisel K, Ludwig H, Bahlis N, Banos A, Tiab M, Delforge M, Cavenagh J, Geraldes C, Lee JJ, Chen C, Oriol A, de la Rubia J, Qiu L, White DJ, Binder D, Anderson K, Fermand JP, Moreau P, Attal M, Knight R, Chen G, Van Oostendorp J, Jacques C, Ervin-Haynes A, Avet-Loiseau H, Hulin C, Facon T, FIRST Trial Team (2014). Lenalidomide and dexamethasone in transplant-ineligible patients with myeloma. N Engl J Med.

[4] Mateos MV, Dimopoulos MA, Cavo M, Suzuki K, Jakubowiak A, Knop S, Doyen C, Lucio P, Nagy Z, Kaplan P, Pour L, Cook M, Grosicki S, Crepaldi A, Liberati AM, Campbell P, Shelekhova T, Yoon SS, Iosava G, Fujisaki T, Garg M, Chiu C, Wang J, Carson R, Crist W, Deraedt W, Nguyen H, Qi M, San-Miguel J, ALCYONE Trial Investigators (2018). Daratumumab plus bortezomib, melphalan, and prednisone for untreated myeloma. N Engl J Med.

[5] Fujisaki T, Ishikawa T, Takamatsu H, Suzuki K, Min CK, Lee JH, Wang J, Carson R, Crist W, Qi M, Nagafuji K (2019). Daratumumab plus bortezomib, melphalan, and prednisone in East Asian patients with non-transplant multiple myeloma: subanalysis of the randomized phase 3 ALCYONE trial. Ann Hematol.

[6] Facon T, Kumar S, Plesner T, Orlowski RZ, Moreau P, Bahlis N, Basu S, Nahi H, Hulin C, Quach H, Goldschmidt H, O’Dwyer M, Perrot A, Venner CP, Weisel K, Mace JR, Raje N, Attal M, Tiab M, Macro M, Frenzel L, Leleu X, Ahmadi T, Chiu C, Wang J, Van Rampelbergh R, Uhlar CM, Kobos R, Qi M, Usmani SZ, MAIA Trial Investigators (2019). Daratumumab plus lenalidomide and dexamethasone for untreated myeloma. N Engl J Med.

[7] Takamatsu H, Iida S, Shibayama H, Shibayama K, Yamazaki H, Suzuki K (2020). Daratumumab, lenalidomide, and dexamethasone in Japanese patients with transplant-ineligible newly diagnosed multiple myeloma: a phase 1b study. Int J Hematol.

[8] Ozaki S, Handa H, Saitoh T, Murakami H, Itagaki M, Asaoku H, Suzuki K, Isoda A, Matsumoto M, Sawamura M, Konishi J, Sunami K, Takezako N, Hagiwara S, Kuroda Y, Chou T, Nagura E, Shimizu K (2015). Trends of survival in patients with multiple myeloma in Japan: a multicenter retrospective collaborative study of the Japanese Society of Myeloma. Blood Cancer J.

[9] International Myeloma Working Group (2003). Criteria for the classification of monoclonal gammopathies, multiple myeloma and related disorders: a report of the International Myeloma Working Group. Br J Haematol.

[10] Rajkumar SV, Dimopoulos MA, Palumbo A, Blade J, Merlini G, Mateos MV, Kumar S, Hillengass J, Kastritis E, Richardson P, Landgren O, Paiva B, Dispenzieri A, Weiss B, LeLeu X, Zweegman S, Lonial S, Rosinol L, Zamagni E, Jagannath S, Sezer O, Kristinsson SY, Caers J, Usmani SZ, Lahuerta JJ, Johnsen HE, Beksac M, Cavo M, Goldschmidt H, Terpos E, Kyle RA, Anderson KC, Durie BG, Miguel JF (2014). International Myeloma Working Group updated criteria for the diagnosis of multiple myeloma. Lancet Oncol.

[11] Durie BG, Harousseau JL, Miguel JS, Bladé J, Barlogie B, Anderson K, Gertz M, Dimopoulos M, Westin J, Sonneveld P, Ludwig H, Gahrton G, Beksac M, Crowley J, Belch A, Boccadaro M, Cavo M, Turesson I, Joshua D, Vesole D, Kyle R, Alexanian R, Tricot G, Attal M, Merlini G, Powles R, Richardson P, Shimizu K, Tosi P, Morgan G, Rajkumar SV, International Myeloma Working Group (2006). International uniform response criteria for multiple myeloma. Leukemia.

[12] Kumar SK, Dispenzieri A, Lacy MQ, Gertz MA, Buadi FK, Pandey S, Kapoor P, Dingli D, Hayman SR, Leung N, Lust J, McCurdy A, Russell SJ, Zeldenrust SR, Kyle RA, Rajkumar SV (2014). Continued improvement in survival in multiple myeloma: changes in early mortality and outcomes in older patients. Leukemia.

[13] Kleber M, Ihorst G, Terhorst M, Koch B, Deschler B, Wäsch R, Engelhardt M (2011). Comorbidity as a prognostic variable in multiple myeloma: comparative evaluation of common comorbidity scores and use of a novel MM-comorbidity score. Blood Cancer J.

[14] Palumbo A, Avet-Loiseau H, Oliva S, Lokhorst HM, Goldschmidt H, Rosinol L, Richardson P, Caltagirone S, Lahuerta JJ, Facon T, Bringhen S, Gay F, Attal M, Passera R, Spencer A, Offidani M, Kumar S, Musto P, Lonial S, Petrucci MT, Orlowski RZ, Zamagni E, Morgan G, Dimopoulos MA, Durie BG, Anderson KC, Sonneveld P, San Miguel J, Cavo M, Rajkumar SV, Moreau P (2015). Revised international staging system for multiple myeloma: a report from international myeloma working group. J Clin Oncol.

[15] Richardson PG, Jacobus SJ, Weller EA, Hassoun H, Lonial S, Raje NS, Medvedova E, McCarthy PL, Libby EN, Voorhees PM, Orlowski RZ, Anderson LD, Zonder JA, Milner CP, Gasparetto C, Agha ME, Khan AM, Hurd DD, Gowin K, Kamble RT, Jagannath S, Nathwani N, Alsina M, Cornell RF, Hashmi H, Campagnaro EL, Andreescu AC, Gentile T, Liedtke M, Godby KN, Cohen AD, Openshaw TH, Pasquini MC, Giralt SA, Kaufman JL, Yee AJ, Scott E, Torka P, Foley A, Fulciniti M, Hebert K, Samur MK, Masone K, Maglio ME, Zeytoonjian AA, Nadeem O, Schlossman RL, Laubach JP, Paba-Prada C, Ghobrial IM, Perrot A, Moreau P, Avet-Loiseau H, Attal M, Anderson KC, Munshi NC, DETERMINATION Investigators (2022). Triplet therapy, transplantation, and maintenance until progression in myeloma. N Engl J Med.

[16] Ozaki S, Harada T, Saitoh T, Shimazaki C, Itagaki M, Asaoku H, Kuroda Y, Chou T, Yoshiki Y, Suzuki K, Murakami H, Hayashi K, Mina R, Palumbo A, Shimizu K, Japanese Society of Myeloma, European Myeloma Network (2014). Survival of multiple myeloma patients aged 65–70 years in the era of novel agents and autologous stem cell transplantation. A multicenter retrospective collaborative study of the Japanese Society of Myeloma and the European Myeloma Network. Acta Haematol.

[17] Avet-Loiseau H, Attal M, Moreau P, Charbonnel C, Garban F, Hulin C, Leyvraz S, Michallet M, Yakoub-Agha I, Garderet L, Marit G, Michaux L, Voillat L, Renaud M, Grosbois B, Guillerm G, Benboubker L, Monconduit M, Thieblemont C, Casassus P, Caillot D, Stoppa AM, Sotto JJ, Wetterwald M, Dumontet C, Fuzibet JG, Azais I, Dorvaux V, Zandecki M, Bataille R, Minvielle S, Harousseau JL, Facon T, Mathiot C (2007). Genetic abnormalities and survival in multiple myeloma: the experience of the Intergroupe Francophone du Myélome. Blood.

[18] Boyd KD, Ross FM, Chiecchio L, Dagrada GP, Konn ZJ, Tapper WJ, Walker BA, Wardell CP, Gregory WM, Szubert AJ, Bell SE, Child JA, Jackson GH, Davies FE, Morgan GJ, NCRI Haematology Oncology Studies Group (2012). A novel prognostic model in myeloma based on co-segregating adverse iFISH lesions and the ISS: analysis of patients treated in the MRC Myeloma IX trial. Leukemia.

[19] Cipkar C, Chen C, Trudel S (2022). Antibodies and bispecifics for multiple myeloma: effective effector therapy. Hematol Am Soc Hematol Educ Program.

[20] Rodriguez-Otero P, San-Miguel JF (2022). Cellular therapy for multiple myeloma: what’s now and what’s next. Hematol Am Soc Hematol Educ Program.

