# Supplementary material for: Primary analysis of a prospective cohort study of Japanese patients with plasma cell neoplasms in the novel drug era (2016–2021)
Source: Int J Hematol. 2024 Mar 29;119(6):707–21. doi: 10.1007/s12185-024-03754-8 (PMC11136844; doi:10.1007/s12185-024-03754-8)
Supplement: Supplementary file 1 — (PPTX 486 KB) [file 12185_2024_3754_MOESM1_ESM.pptx]

## Slide 1
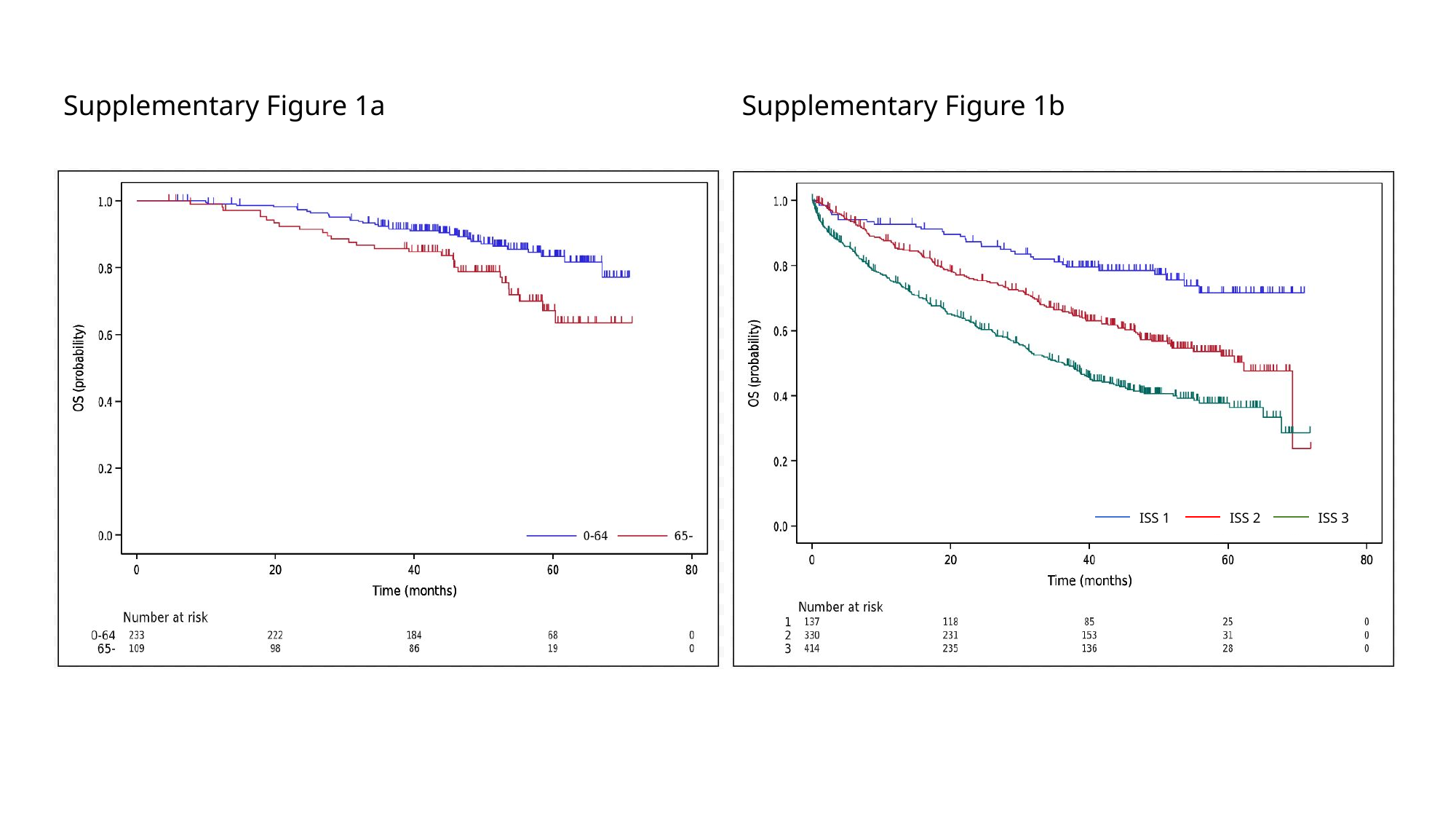

Supplementary Figure 1a
Supplementary Figure 1b
ISS 1
ISS 2
ISS 3

## Slide 2
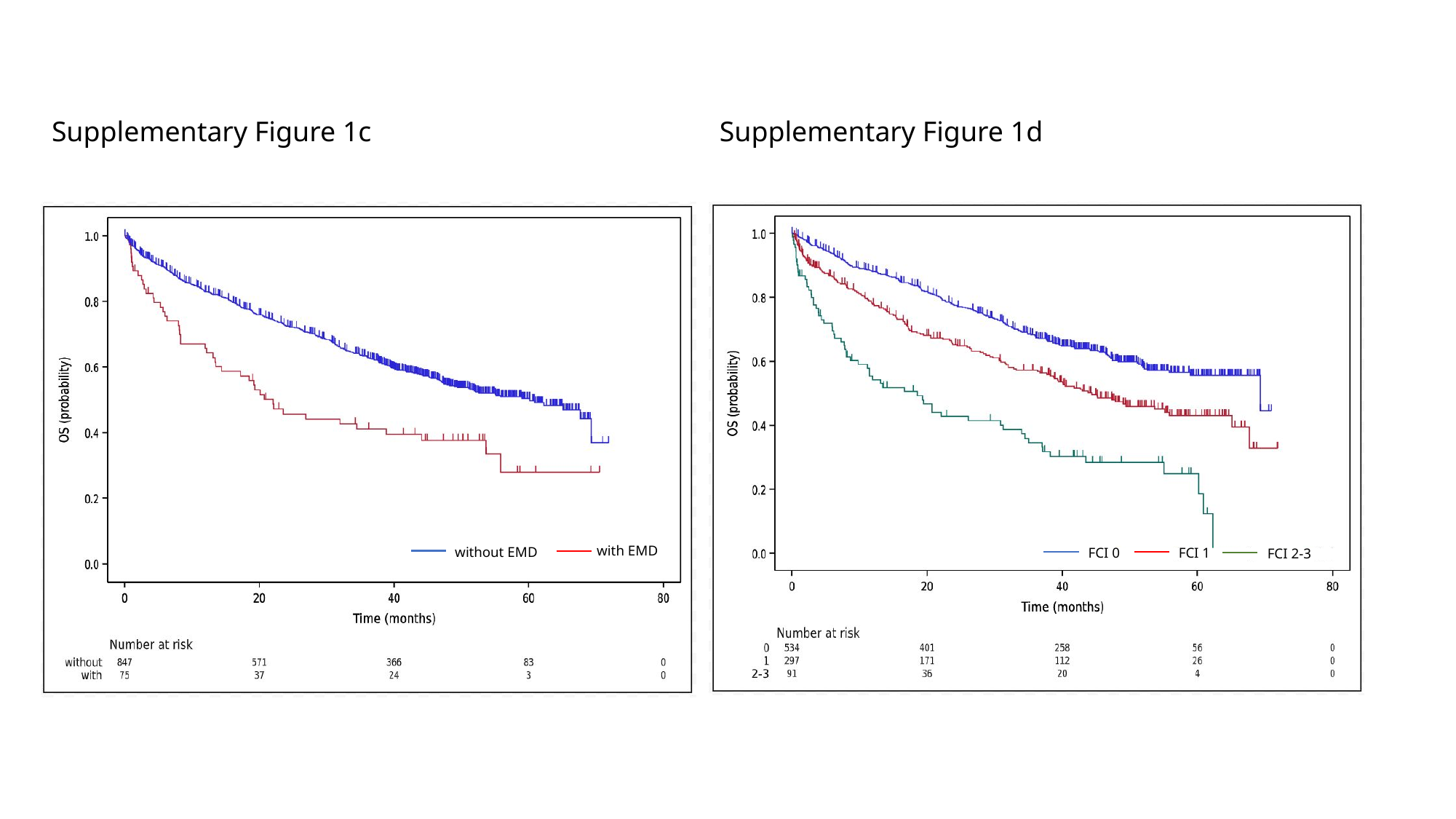

Supplementary Figure 1c
Supplementary Figure 1d
with EMD
without EMD
FCI 0
FCI 1
FCI 2-3

## Slide 3
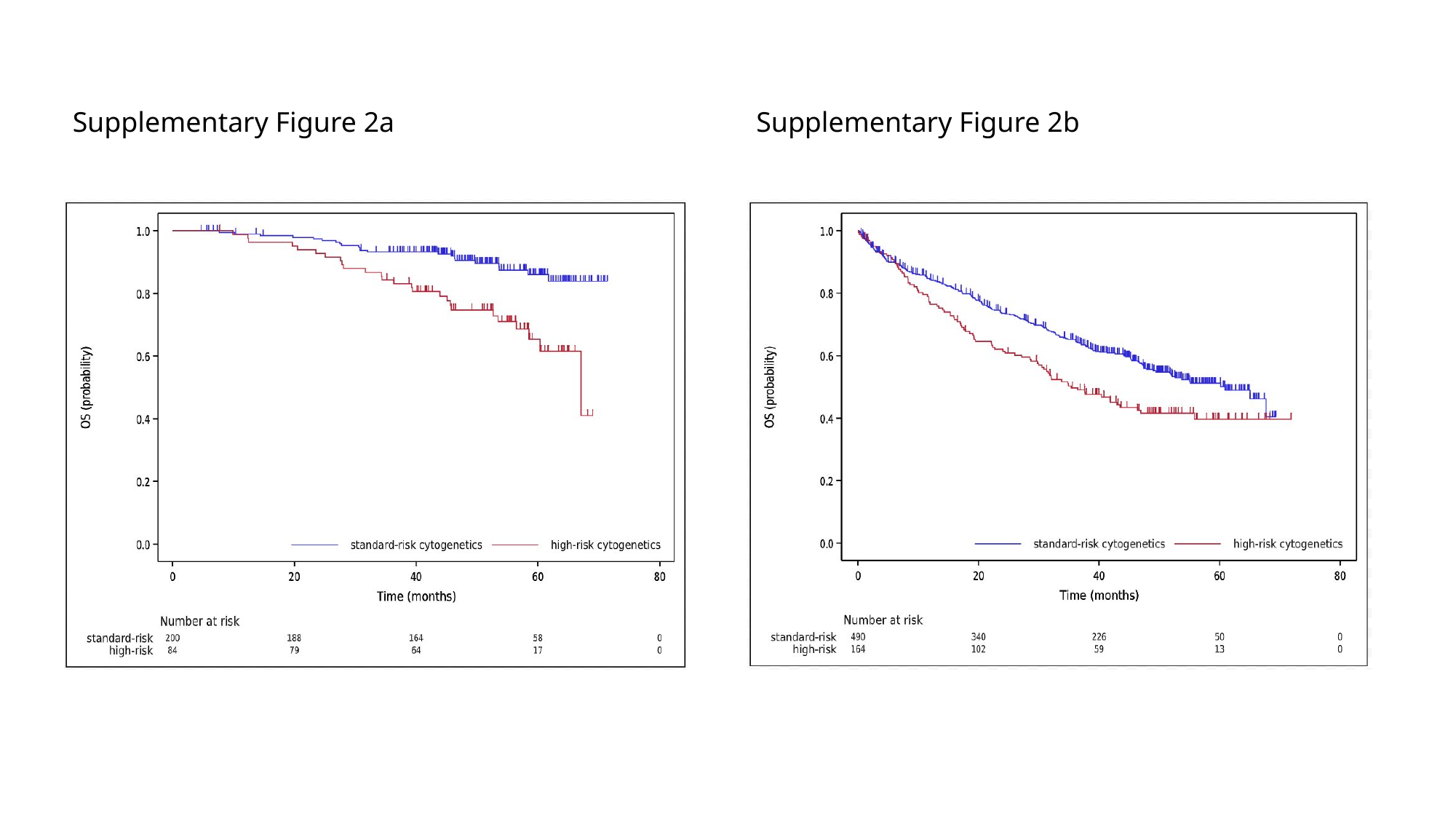

Supplementary Figure 2a
Supplementary Figure 2b

## Slide 4
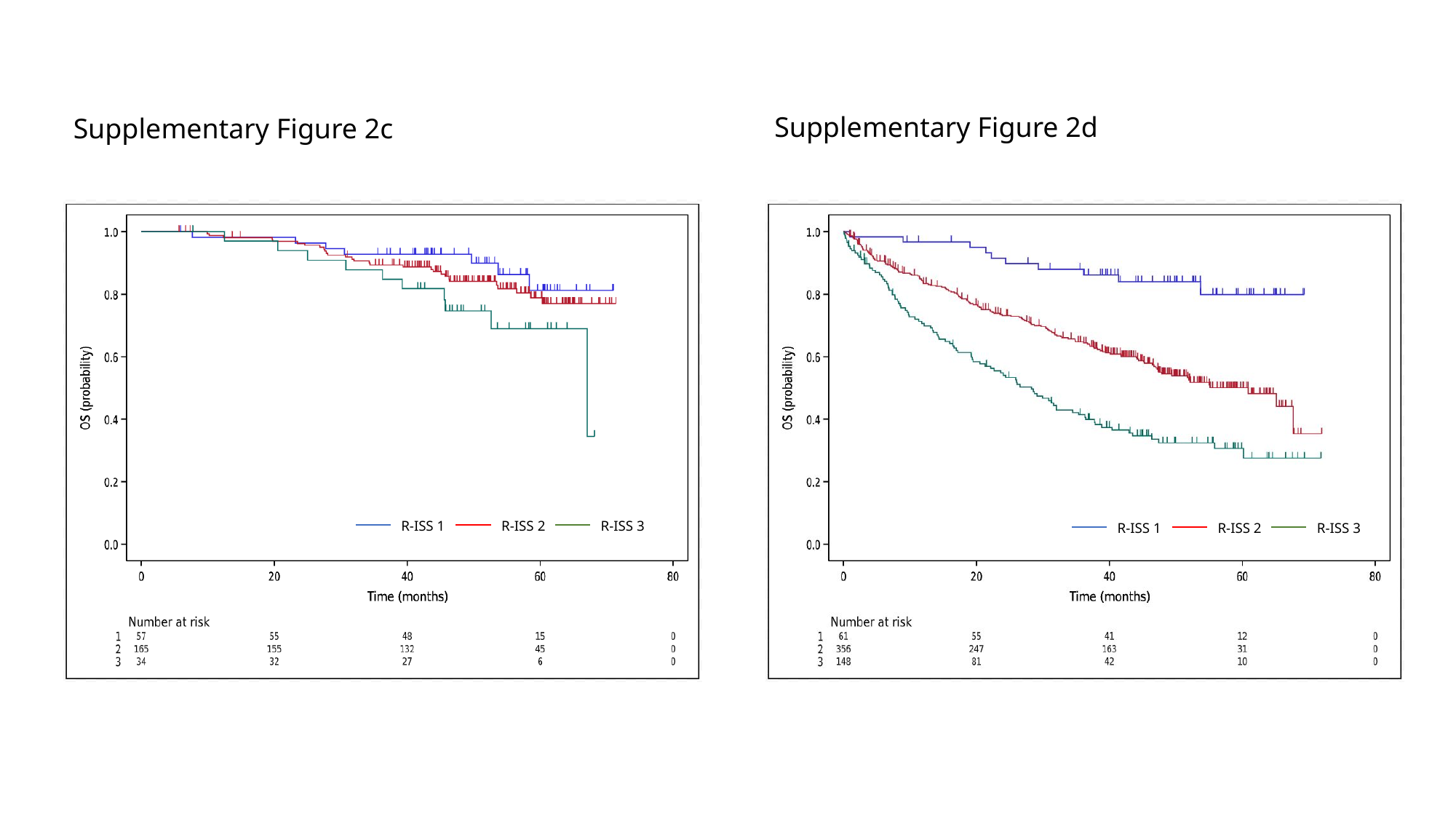

Supplementary Figure 2d
Supplementary Figure 2c
R-ISS 1
R-ISS 2
R-ISS 3
R-ISS 1
R-ISS 2
R-ISS 3

## Slide 5
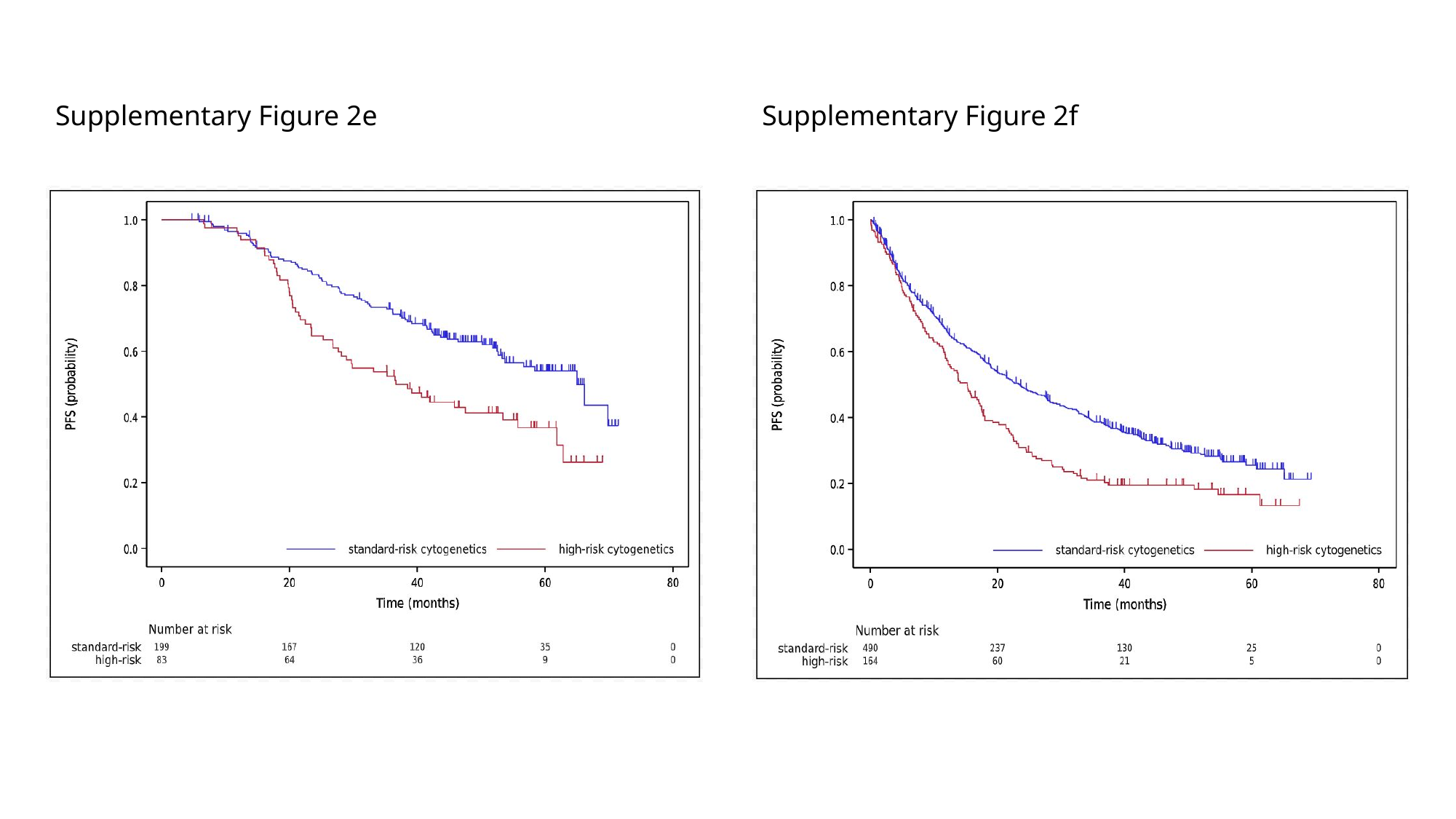

Supplementary Figure 2e
Supplementary Figure 2f
