# Supplementary material for: Primary analysis of a prospective cohort study of Japanese patients with plasma cell neoplasms in the novel drug era (2016–2021)
Source: Int J Hematol. 2024 Mar 29;119(6):707–21. doi: 10.1007/s12185-024-03754-8 (PMC11136844; doi:10.1007/s12185-024-03754-8)
Supplement: Supplementary file 2 — (DOCX 16 KB) [file 12185_2024_3754_MOESM2_ESM.docx]

Supplementary Figure 1. (a) Three-year survival rate of ASCT patients aged 65 or older (red line) was significantly worse than that of those younger than 65 (blue line). (b) Three-year survival rates of non-ASCT patients with ISS 2 (red line) and ISS 3 (green line) were significantly worse than that of those with ISS 1 (blue line). (c) Three-year survival rate of non-ASCT patients with extramedullary tumors (red line) was significantly worse than that without extramedullary tumors (blue line). (d) Three-year survival rate of non-ASCT patients with FCI 2/3 (green line) was significantly worse than that with FCI 0 (blue line).

Supplementary Figure 2. (a) Three-year survival rates of ASCT patients with high-risk cytogenetics (red line) and standard-risk cytogenetics (blue line) were 84.3% (95% CI: 74.6-90.6) and 93.2% (95% CI: 88.6-90.6), respectively. (b) Three-year survival rates of non-ASCT patients with high-risk cytogenetics (red line) and standard-risk cytogenetics (blue line) were 49.7% (95%CI: 41.6-57.2) and 65.2% (95%CI: 60.6-69.4), respectively. (c) Three-year survival rates of ASCT patients with R-ISS 1 (blue line), 2 (red line), 3 (green line) were 92.9% (95%CI: 82.1-97.3), 89.4% (95%CI: 83.5-93.3) and 87.9% (95%CI: 70.9-95.3), respectively. (d) Three-year survival rates of non-ASCT patients with R-ISS 1 (blue line), 2 (red line), 3 (green line) were 88.1% (95%CI: 76.5-94.1), 64.7% (95%CI: 59.2-69.6) and 41.4% (95%CI: 33.2-49.5), respectively. (e) Three-year PFS rates of ASCT patients with high-risk cytogenetics (red line) and standard-risk cytogenetics (blue line) were 52.4% (95%CI: 41.1-62.6) and 72.8% (95%CI: 65.9-78.6), respectively. (f) Three-year PFS rates of non-ASCT patients with high-risk cytogenetics (red line) and standard-risk cytogenetics (blue line) were 20.9% (95%CI: 15.0-27.6) and 38.6% (95%CI: 34.1-43.1), respectively.

(Supplementary Method S1) OS was the time from start of initial treatment to death of any cause. Survivors were censored on the last date of follow-up. PFS was the time from start of initial treatment to the earlier date of disease progression, or death of any cause. Survivors without disease progression were censored on the date the patient was confirmed to be progression-free. TNT was the duration between start of initial treatment and start of the next line treatment, death of any cause. Survivors without the next line treatment were censored on the last date of follow-up. TFI was the duration between the date of last dose of initial treatment and the date of the first dose of the next line of therapy, or death of any cause. Survivors without next line of treatment were censored on the last date of follow-up.

Supplementary list (the list of the institutions attending this study)

Department of Hematology, Hiroshima Red Cross Hospital

Department of Hematology, Gunma University

Department of Hematology, NHO Tokyo Medical Center

Department of Hematology and Oncology, Nagoya City University

Department of Hematology, NHO Shibukawa Medical Center

Department of Hematology, Japanese Red Cross Medical Center

Department of Hematology, Toyonaka Municipal Hospital

Department of Hematology, Sapporo Hokuyu Hospital

Department of Hematology and Rheumatology, Kagoshima University

Department of Hematology, Japanese Red Cross Wakayama Medical Center

Department of Hematology and Oncology, Toyohashi Municipal Hospital

Department of Hematology and Oncology, Osaka University

Department of Hematology, JCHO Kyoto Kuramaguchi Medical Center

Department of Hematology, Saitama Medical Center, Saitama Medical University

Department of Hematology, Yamanashi Prefecture Central Hospital

Department of Hematology, Keio University

Department of Hematology, Sasebo City General Hospital

Department of Hematology, NHO Okayama Medical Center

Department of Hematology, Fujioka General Hospital

Department of Hematology and Oncology, Dokkyo Medical University

Department of Hematology, Juntendo University

Department of Hematology, Miyagi Cancer Center

Department of the first Internal Medicine, Gifu University

Department of Hematology, Oita Prefecture Hospital

Department of Oncology and Hematology, Shimane University

Department of Hematology, Hyogo Cancer Center

Department of Hematology, Japanese Red Cross Kyoto Second Medical Center

Department of Hematology and Oncology, Nagoya Municipal Western Medical Center

Department of Hematology, Kanazawa University

Department of Hematology, Rheumatology, and Infectious diseases, Yokohama City University

Department of Hematology, Oita Koseiren Tsurumi Hospital

Department of Hematology, Tokushima Prefecture Central Hospital

Department of Hematology, PL Hospital

Department of Hematology, Shiga Medical University

Department of Hematology, Tsukuba University

Department of Hematology, Toyota Kosei Hospital

Department of the third Internal Medicine, Yamagata University

Department of Internal Medicine, Toyama Prefecture Central Hospital

Department of Hematology, Fukushima Prefecture Medical University

Department of Hematology and Oncology, Tosei General Hospital

Department of Hematology, Nagasaki University

Department of Hematology and Immunology, Tohoku University

Department of Hematology, International Medical Research Center

Department of Internal Medicine, Naha Municipal Hospital

Department of Hematology, Japanese Red Cross Toyama Medical Center

Department of Hematology and Transfusion, Kochi Medical Center

Department of Hematology and Oncology, Kyoto Prefecture University of Medicine

Department of Hematology, Fujita Medical University

Department of Hematology and Oncology, Kyushu University

Department of Hematology, Eiju General Hospital

Department of Hematology, Aizu Medical Center, Fukushima Medical University

Department of Hematology, Japanese Red Cross Yokohama Minato Medical Center

Department of Hematology, Kashiwazaki General Medical Center

Department of Hematology, Aomori Prefecture Central Hospital

Department of Hematology, Toyama University

Department of Hematology, Chiba Municipal Aoba Hospital

Department of Hematology, NHO Kyushu Medical Center

Department of Hematology, NHO Kyushu Cancer Center

Department of Hematology, Sapporo Kosei General Hospital

Department of Hematology, Tonami Municipal General Hospital

Department of Hematology and Oncology, Tokyo University

Department of Hematology and Rheumatology, Kindai University

Department of Hematology, Nagoya University

Department of Hematology, JCHO Sapporo Hokusin Hospital

Department of Oncology, Hematology and Infectious diseases, Fukuoka University

Department of Hematology, Mie University

Department of Hematology and Oncology, Hachioji Hospital, Tokai University
